# Predicting uninformative prostate magnetic resonance imaging sequences: a hypothesis-generating pilot study

**DOI:** 10.1590/0100-3984.2025.0007

**Published:** 2025-07-17

**Authors:** Negar Firoozeh, Spencer C. Behr, Antonio C. Westphalen

**Affiliations:** 1 University of Washington, Seattle, WA, USA; 2 University of California, San Francisco, CA, USA

**Keywords:** Prostate/diagnostic imaging, Prostatic neoplasms, Magnetic resonance imaging/methods, Próstata/diagnóstico por imagem, Neoplasias da próstata, Ressonância magnética/métodos

## Abstract

**Objective:**

To determine the proportion of men with completely negative multiparametric magnetic resonance imaging (MRI) scans and which individual sequence-T2-weighted imaging (T2WI) or diffusion-weighted imaging (DWI)-best predicts an overall negative examination result.

**Materials and Methods:**

This was a single-center retrospective study evaluating 492 MRI scans compliant with Prostate Imaging Reporting and Data System (PI-RADS), version 2.1. Radiology reports described the absence of lesions or suspicious lesions with PI-RADS scores of 3-5, signifying positive T2WI or DWI results. Positivity on a dynamic contrast-enhanced (DCE) study was determined by early or simultaneous focal enhancement consistent with lesions on T2WI or DWI. All scans reported as negative were prospectively reviewed to ensure that each sequence truly met the criteria for negativity according to the PI-RADS guidelines. Descriptive statistics were employed to summarize the data, and the chi-square test was employed to assess the relationship between a negative T2WI result and a negative DWI/DCE result, as well as that between a negative DWI result and a negative DWI/DCE result, with logistic regression models identifying predictors of such combined results.

**Results:**

Among the patients evaluated, approximately one-third of those with suspected prostate cancer and 10% of those with known cancer could have concluded their examination after a single negative sequence. A negative T2WI result predicted negative DWI/DCE findings in 62.4% of scans (95% CI: 55.3-68.9), with an odds ratio of 245.3 (*p* < 0.001). Similarly, a negative DWI result predicted negative T2WI/DCE findings in 88.9% of scans (95% CI: 83.1-92.7) with an odds ratio of 76.4 (*p* < 0.001). These associations remained robust after adjustment for age, prostate-specific antigen level, prostate-specific antigen density, cancer status, and radiologist.

**Conclusion:**

Findings from T2WI or DWI may serve as preliminary indicators for the subsequent diagnostic yield of other sequences, with DWI appearing to hold a slight advantage. Although the accuracy of this approach is not yet sufficient for clinical implementation, these results are promising and merit further investigation.

## Abstract (pt)

**Objetivo:**

Determinar a proporção de homens com exames de ressonância magnética (RM) multiparamétrica completamente negativos e qual sequência individual - imagem ponderada em T2 ou imagem ponderada em difusão (IPD) - prevê melhor um resultado geral negativo no exame.

**Materiais e Métodos:**

Este foi um estudo retrospectivo unicêntrico avaliando 492 exames de RM compatíveis com o Prostate Imaging Reporting and Data System (PI-RADS), versão 2.1. Os relatórios de radiologia descreveram a ausência de lesões ou lesões suspeitas com pontuações PI-RADS de 3 a 5, significando resultados positivos em T2 ou IPD. A positividade em um estudo de contraste dinâmico (*dynamic contrast-enhanced -* DCE) foi determinada pelo realce focal precoce ou simultâneo consistente com lesões em T2 ou IPD. Todos os exames relatados como negativos foram revisados prospectivamente para garantir que cada sequência realmente atendesse aos critérios de negatividade de acordo com as diretrizes PI-RADS. Estatísticas descritivas foram empregadas para resumir os dados, e o teste do qui-quadrado foi empregado para avaliar a relação entre um resultado negativo em T2 e um resultado negativo em IPD/DCE, bem como entre um resultado negativo em IPD e um resultado negativo em IPD/DCE, com modelos de regressão logística identificando preditores de tais resultados combinados.

**Resultados:**

Entre os pacientes avaliados, aproximadamente um terço dos com suspeita de câncer de próstata e 10% dos com câncer conhecido poderiam ter concluído seu exame após uma única sequência negativa. Um resultado negativo em T2 previu achados negativos em IPD/DCE em 62,4% dos exames (IC 95%: 55,3-68,9), com uma razão de chances de 245,3 (*p* < 0,001). Da mesma forma, um resultado negativo em IPD previu achados negativos em T2/DCE em 88,9% dos exames (IC 95%: 83,1-92,7), com uma razão de chances de 76,4 (*p* < 0,001). Essas associações permaneceram robustas após ajuste para idade, nível de antígeno prostático específico, densidade de antígeno prostático específico, *status* do câncer e radiologista.

**Conclusão:**

Os achados em T2 ou IPD podem servir como indicadores preliminares para o rendimento diagnóstico subsequente de outras sequências, com IPD parecendo apresentar uma ligeira vantagem. Embora a precisão dessa abordagem ainda não seja suficiente para implementação clínica, esses resultados são promissores e merecem investigação mais aprofundada.

## INTRODUCTION

The increasing demand for magnetic resonance imaging (MRI) services is multifactorial, reflecting longstanding trends documented as early as 2009^(1)^, and has surged further in the wake of the coronavirus disease 2019 pandemic^(2^,^3)^. Meanwhile, this increasing volume of examinations, coupled with limited radiologist availability and evolving workplace expectations^(4^-^6)^, underscores the need for more streamlined imaging workflows to ensure timely, high-quality patient care. One strategy to enhance efficiency is by shortening the duration of MRI examinations, commonly achieved by reducing the number of MRI sequences acquired, thus saving scanner time and personnel resources^(7^-^9)^. This approach is particularly beneficial in prostate cancer evaluation, in which MRI-which typically includes T2-weighted imaging (T2WI), diffusion-weighted imaging (DWI), and dynamic contrast-enhanced (DCE) sequences-plays a pivotal role. It is widely employed to assess men with suspected or known prostate cancer, especially in the context of the MRI pathway, in which it serves as a triage tool prior to biopsy in men with elevated serum prostate-specific antigen (PSA) levels^(10)^. Consequently, prostate volumes on MRI have increased substantially, exacerbating existing challenges in access and workflow^(11^-^13)^.

To address these concerns, current discussions revolve around the use of biparametric MRI protocols, which exclude the use of contrast agents to reduce examination time^(14^-^16)^. Another potential approach draws inspiration from adrenal nodule imaging, in which the initial unenhanced computed tomography scan is reviewed before deciding whether contrast-enhanced imaging will provide valuable additional information^(17)^. Translating this concept to prostate MRI, the ideal scenario would be a “smart abbreviated protocol” in which artificial intelligence (AI) models assess the first sequence acquired, such as T2WI or DWI, in real time and determine whether the remainder of the examination can be safely omitted^(18)^. This would allow early termination of scans unlikely to reveal suspicious lesions, significantly improving throughput without compromising diagnostic accuracy^(19)^.

As a necessary first step toward this goal, the present study aims to provide proof of concept for such an approach. Specifically, we seek to determine the proportion of men with completely negative multiparametric MRI scans, as well as which individual sequence (T2WI or DWI) best predicts an overall negative examination.

## MATERIALS AND METHODS

This was a Health Insurance Portability and Accountability Act-compliant retrospective cross-sectional study approved by our institutional review board. Because of the retrospective nature of the study, the requirement for informed consent was waived.

### Patient selection

**Figure 1:**
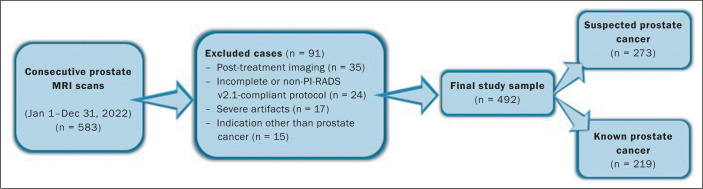
Flowchart illustrating the patient selection process.

All prostate MRI scans obtained from January 1, 2022, to December 31, 2022 were found by querying our picture archiving and communication system. For patients who underwent multiple scans, we included only the first scan in our analysis, which resulted in an initial pool of 583 examinations. Our exclusion criteria were as follows: post-treatment imaging; substantial artifact from hip replacements; other severe imaging artifact(s); imaging protocol not meeting Prostate Imaging Reporting and Data System (PI-RADS), version 2.1, standards or incomplete examination; and indication other than prostate cancer. Following these criteria, we obtained a final sample of 492 prostate MRI scans that were in full compliance with the PI-RADS guidelines and included T2WI, DWI, and DCE sequences^(20)^, as illustrated in Figure 1. All images were acquired in 3.0-T MRI scanners (Ingenia; Philips Healthcare, Best, the Netherlands), with a multichannel surface coil. In all examinations, a gadolinium-based contrast agent (gadoteridol) was administered intravenously (0.1 mmol/kg; 3 mL/s). The MRI protocol is shown in Table 1.

**Table 1:** MRI protocols.

Sequence	Type	Plane	Slice (mm)	Gap (mm)	Phase	Resolution (mm)	FOV (mm)	Coverage	Comments
T2WI	2D SS	Sagittal	4.0	0	AP	1.52 x 1.5	132 x133	Base of penis to above seminal vesicles, entire prostate	Check if air in the rectum
T2WI	2D TSE	Axial	3.2	0	RL	0.5 x 0.8	160 x 160	Prostate and seminal vesicles, including membranous urethra	Check for motion, repeat if necessary
DWI (b = 0, 800)	2D SS-EPI	Axial	3.0	0	AP	2.5 x 3.06	96x39	Prostate and seminal vesicles, including membranous urethra	Generate synthetic b-value of 1,400
T2WI	2D TSE	Sagittal	3.2	0	AP	0.5 x 0.8	200 x 200	Base of penis to above seminal vesicles, entire prostate	
T2WI	2D TSE	Coronal	3.2	0	RL	0.5 x 0.82	200 x 200	Base of penis to above seminal vesicles, entire prostate	
T1WI	2D TSE	Axial	3.2	0	RL	0.9 x 0.9	160 x 160	Prostate and seminal vesicles, including membranous urethra	
DWI (b = 0, 1,400)	2D SS-EPI	Axial	3.0	0	RL	2.5 x 3.06	96x39	Prostate and seminal vesicles, including membranous urethra	Generate synthetic b-value of 1,400
T1WI	3DGRE	Axial	2.0	0	AP	1.6 x 1.8	200 x160	Prostate and seminal vesicles, including membranous urethra	12 time points; temporal resolution = 10 s
T1WI	3D mDIXON	Axial	2.6	0	AP	1.5 x 1.7	360 x 270	From aortic bifurcation to below the scrotum	Breath-hold

FOV, field of view; 2D, two-dimensional; SS, single-shot; AP, anteroposterior; TSE, turbo spin-echo; RL, right-to-left; EPI, echo-planar imaging; 3D, three-dimensional; GRE, gradient-echo; mDIXON, modified DIXON.

### Data collection and image interpretation

One of the authors collected patient information from the electronic medical records. The following data were included: patient age; serum PSA and PSA density at the time of imaging; cancer status at the time of imaging (suspected versus biopsy-proven); management at the time of imaging (new diagnosis versus active surveillance); T2WI PI-RADS scores; DWI PI-RADS scores; DCE results; and the identity of the radiologist evaluating the MRI. Clinical MRI interpretations were conducted by a group of 13 abdominal radiologists with 2-22 years of post-fellowship experience. Original structured reports described either no suspicious lesions or enumerated suspicious lesions and classified them according to the PI-RADS guidelines.

On T2WI and DWI, a PI-RADS of score 3, 4, or 5 denoted a positive result, whereas a score of 1 or 2 denoted a negative result. For DCE, positive results were defined by the occurrence of early or contemporaneous focal enhancement that aligned with a lesion on T2WI or DWI. Any different pattern of enhancement was deemed a negative outcome.

One of the authors prospectively reviewed all MRI scans reported as negative, to ensure that each sequence (i.e., T2WI, DWI, and DCE) truly met the criteria for negativity according to the PI-RADS guidelines.

### Statistical analysis

Each examination represented a unit of analysis in our study, and all analyses were conducted on a per-patient basis. For the purposes of this study, an examination was classified as positive if any sequence showed at least one suspicious finding, regardless of the number or location of lesions; conversely, examinations were considered negative only when all three sequences (T2WI, DWI, and DCE) were negative. This binary classification reflects the clinical decision-making context, in which the presence of any lesion typically prompts further evaluation, such as targeted biopsy.

Data were summarized using descriptive statistics and measures of dispersion. Two separate chi-square analyses were performed: one examined the relationship between T2WI results and the combined DWI/DCE results, and the other assessed the relationship between DWI results and the combined T2WI/DCE results. Analyses were stratified by the clinical indication: suspected prostate cancer versus follow-up of known prostate cancer. Similarly, univariate and multivariate logistic regression models were employed to identify predictors of two distinct outcomes: the DWI and DCE sequences both being negative; and the T2WI and DCE sequences both being negative. In each model, the dependent variable was binary: either both sequences were negative or at least one was positive. For the first outcome (negative DWI/DCE), T2WI results served as the primary predictor of interest, whereas DWI results were the primary predictor for the second outcome (negative T2WI/DCE). Additional potential predictors tested included age, serum PSA, PSA density, cancer status, and the interpreting radiologist. All statistical analyses were performed using the Stata statistical software package, version 18.0 (StataCorp LLC, College Station, TX, USA), with a two-tailed significance level set at α = 0.05.

## RESULTS

A total of 492 prostate MRI scans (492 unique patients) were included in this study. The mean patient age was 66.4 ± 8.5 years. Median serum PSA and PSA density were 6.7 ng/mL (interquartile range: 5.0-10.1 ng/mL) and 0.13 ng/mL/cm^3^ (interquartile range: 0.09-0.20 ng/mL/cm^3^). Of the 492 patients evaluated, 273 (55.5%) had suspected prostate cancer and the remaining 219 (44.5%) had biopsy-proven prostate cancer. Of the 219 patients with known prostate cancer, 133 (60.7%) were under active surveillance and 86 (39.2%) had newly diagnosed cancer. Among the patients with positive biopsy results, the cancer was categorized, as defined by the International Society of Urological Pathology, as grade group 1 in 30.2%, grade group 2 in 43.8%, grade group 3 in 18.1%, grade group 4 in 3.1%, and grade group 5 in 4.5%.

Table 2 summarizes the distribution of T2WI, DWI, and DCE results for all 492 scans. Notably, entirely negative MRI examinations (i.e., examinations in which T2WI, DWI, and DCE were negative) were seen in 24.6% (95% CI: 20.8-28.4%) of the patients. When stratified by clinical indication, this proportion was 33.0% (95% CI: 27.6-72.4%) among the patients with suspected prostate cancer and 14.2% (95% CI: 10.1-19.5) among those with known prostate cancer.

**Table 2:** Proportions of negative findings across T2WI, DWI, and DCE sequences.

Sequence	Entire cohort (N = 492)	
	n (%)	95% Cl
T2WI	194 (39.4)	35.1-43.8
DWI	136 (27.6)	23.7-31.6
DCE	207 (42.1)	37.7-46.4
Combined DWI/DCE	123 (25.0)	21.2-28.8
Combined T2WI/DCE	155 (31.5)	27.4-35.6
All three	121 (24.6)	20.8-28.4
	Suspected prostate cancer (n = 273)	
Sequence	n (%)	95% Cl
T2Wla	142 (52.0)	46.1-57.9
DWI	100 (36.6)	31.1-42.5
DCE	143 (52.4)	46.4-58.3
Combined DWI/DCE	92 (33.7)	28.3-39.5
Combined T2WI/DCE	113 (41.4)	35.7-47.4
All three	90 (33.0)	27.6-72.4
	Known prostate cancer (n = 219)	
Sequence	n (%)	95% Cl
T2WI	52 (23.7)	18.5-29.9
DWI	36 (16.4)	12.1-22.0
DCE	64 (29.2)	23.6-35.6
Combined DWI/DCE	31(14.2)	10.1-19.5
Combined T2WI/DCE	42 (19.2)	14.5-25.0
All three	31(14.2)	10.1-19.5

Of the scans with a negative T2WI result, 62.4% (95% CI: 55.3-68.9) also had negative combined DWI/DCE findings (*p* < 0.001), whereas positive combined DWI/DCE findings were seen in 99.3% (95% CI: 97.3-99.8) of those with a positive T2WI result. Table 3 presents these results stratified by prostate cancer status. However, 88.9% (95% CI: 83.1-92.7) of scans with a negative DWI result also showed negative combined T2WI/DCE findings (*p* < 0.001), whereas 90.4% (95% CI: 87.0-93.1) of scans with a positive DWI result demonstrated positive combined T2WI/DCE findings. Table 4 provides these results stratified by cancer status.

**Table 3:** Correlation between T2WI and combined DWI/DCE results, by prostate cancer status.

Combined DWI/DCE	Suspected prostate cancer (n = 273)		
	Negative T2WI	Positive T2WI	P-value
Negative			
n	90	2	
% (95% Cl)	63.4 (55.1-70.9)	1.5 (0.3-5.9)	
Positive			
n	52	129	
% (95% Cl)	36.6 (29.1-44.9)	98.5 (94.1-99.6)	
Total, n (%)	142 (52.0)	131 (48.0)	< 0.001
	Known prostate cancer (n = 219)		
Combined DWI/DCE	Negative T2WI	Positive T2WI	P-value
Negative			
n	31	0	
% (95% Cl)	59.6 (45.8-72.0)	0(-)	
Positive			
n	21	167	
% (95% Cl)	40.4 (28.0-54.2)	100 (-)	
Total, n (%)	52 (9.6)	167 (76.3)	< 0.001

**Table 4:** Correlation between DWI and combined T2WI/DCE results, by prostate cancer status.

Combined T2WI/DCE	Suspected prostate cancer (n = 273)		
	Negative DWI	Positive DWI	P-value
Negative			
n	90	23	
% (95% Cl)	90.0 (82.4-94.5)	13.3 (9.0-19.2)	
Positive			
n	10	150	
% (95% Cl)	10.0 (5.5-17.6)	86.7 (80.1-91.0)	
Total, n (%)	100 (36.6)	173 (63.4)	0.000
	Known prostate cancer (n = 219)		
Combined T2WI/DCE	Negative DWI	Positive DWI	P-value
Negative			
n	31	11	
% (95% Cl)	86.1 (70.6-94.1)	6.0 (3.3-10.6)	
Positive			
n	5	172	
% (95% Cl)	13.9 (5.9-29.4)	94.0 (89.4-96.7)	
Total, n (%)	36 (16.4)	183 (83.6)	0.000

**Figure 2:**
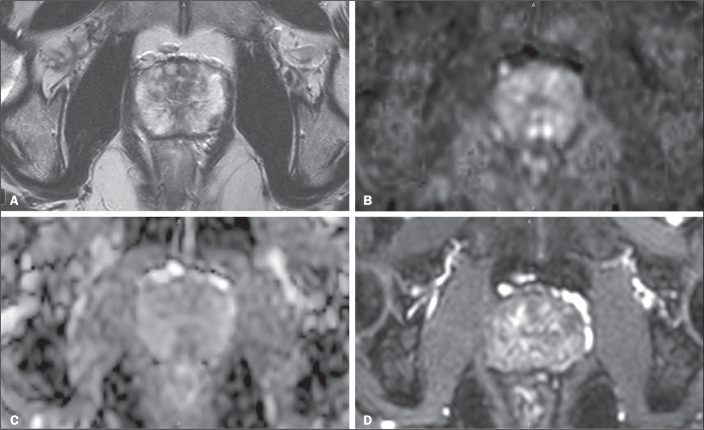
Multiparametric prostate MRI from a 62-year-old patient with suspected prostate cancer (serum PSA: 4.3 ng/mL; prostate volume: 27 cm^3^; PSA density: 0.16 ng/mL/cm^3^). All sequences were negative for suspicious findings according to PI-RADS criteria: A, axial T2-weighted image; B, high b-value diffusion-weighted imaging (b = 1,400 s/mm^2^); C, apparent diffusion coefficient map; D, DCE image.

Logistic regression findings (Tables 5 and 6) indicate that the initial MRI sequence is a strong predictor of whether the remaining two sequences will be negative. Specifically, Table 5 shows that T2WI results predict the likelihood of negative combined DWI/DCE findings, and Table 6 demonstrates that DWI results similarly predict negative combined T2WI/DCE findings. In contrast, other variables, including age, serum PSA, PSA density, cancer status, and the interpreting radiologist, do not appear to have a meaningful impact on the predictive power of these models. A representative example of a patient with a completely negative multiparametric MRI examination is shown in Figure 2, demonstrating negative T2WI-weighted, high b-value DWI, apparent diffusion coefficient map, and DCE images in a 62-year-old patient with suspected prostate cancer (serum PSA, 4.3 ng/mL; prostate volume, 27 cm^3^; and PSA density, 0.16 ng/mL/cm^3^).

**Table 5:** Logistic regression for combined DWI/DCE results.

			Univariate	model	
Variable	OR	SE	z	*P*	95% Cl
T2WI	245.32	177.81	7.59	0.000	59.26-1015.50
_cons	0.60	0.09	-3.41	0.001	0.45-0.81
LR chi^^2^^(l) = 272.39	*P* > chi^^2^^ = 0.0		000 Pseudo *R^2^ =*		0.49
			Multivaris	ite model	
Variable	OR	SE	z	*P*	95% Cl
T2WI	603.78	634.29	6.10	0.000	77.03-4732.51
Age	0.96	0.02	-2.19	0.03	0.92-0.99
PSA density	0.95	0.08	-0.64	0.52	0.82-1.11
Indication: proven	1.52	0.55	1.16	0.25	0.75-3.08
Radiologist 2	0.73	0.98	-0.23	0.82	0.05-10.23
Radiologist: 3	(empty)				
Radiologist: 5	0.25	0.33	-1.04	0.30	0.02-3.35
Radiologist 6	0.56	0.89	-0.36	0.72	0.03-12.73
Radiologist 7	0.35	0.49	-0.76	0.45	0.02-5.22
Radiologist: 8	0.01	0.02	-2.51	0.01	0.00-0.37
Radiologist: 9	0.02	0.08	-1.08	0.28	0.00-20.79
Radiologist: 10	(empty)				
Radiologist: 11	0.39	0.50	-0.73	0.47	0.03-4.84
Radiologist: 12	0.03	0.11	-0.92	0.36	0.00-61.78
Radiologist: 13	0.27	0.34	-1.05	0.30	0.02-3.11
_cons	0.60	0.09	-3.41	0.001	0.45-0.81
LR chi^^2^^(13) = 273.12	*P* > chi	^2^ = 0.00	00	Pseudo *R^2^* = 0.55	

_cons, constant/intercept term; LR, likelihood ratio.

**Table 6:** Logistic regression for combined T2WI/DCE results.

Variable	Univariate model				
	OR	SE	*z*	*P*	95% Cl
DWI	76.40	25.04	13.23	0.000	40.18-145.24
_cons	0.12	0.03	-7.63	0.000	0.07-0.21
LR ch¡^^2^^(l) = 294.34	*P>*	chi^^2^^ = 0.0000 Pseudo *R^2^ =*			0.48
			Multivariate model		
Variable	OR	SE	*z*	*p*	95% Cl
DWI	99.06	38.51	11.82	0.000	46.23-212.22
Age	1.05	0.02	2.50	0.01	1.01-1.09
PSA density	0.99	0.08	-0.15	0.88	0.85-1.15
Indication: proven	1.84	0.64	1.77	0.08	0.94-3.63
Radiologist: 2	1.57	1.94	0.37	0.71	0.14-17.56
Radiologist: 3	1	(empty)			
Radiologist: 5	1.11	1.26	0.09	0.93	0.12-10.33
Radiologist: 6	3.28	4.62	0.84	0.40	0.21-52.02
Radiologist: 7	1.05	1.21	0.04	0.97	0.11-10.07
Radiologist: 8	7.11	9.51	1.47	0.14	0.52-97.94
Radiologist: 9	3.32	8.12	0.49	0.62	0.03-399.80
Radiologist: 10	1	(empty)			
Radiologist: 11	1.33	1.49	0.25	0.80	0.15-11.94
Radiologist: 12	1.66	4.36	0.19	0.85	0.01-281.79
Radiologist: 13	1.61	1.71	0.44	0.66	0.20-12.91
_cons	0.002	0.004	-3.42	0.001	0.00-0.07
LR chi^^2^^(13) = 286.66	*P>*	chi^^2^^ = 0.0000		Pseudo *R^2^* = 0.53	

_cons, constant/intercept term; LR, likelihood ratio.

## DISCUSSION

Our findings indicate that the initial MRI sequence (T2WI or DWI) can reliably predict the likelihood that the remaining sequences (DWI/DCE or T2WI/DCE, respectively) will also be negative. In this context, “negative” refers to sequences that do not reveal suspicious findings and therefore do not add diagnostic value beyond what is already available from the initial sequence. Negative findings on the initial T2WI or DWI sequence were highly indicative of an overall negative examination, with statistical models identifying these sequences as the strongest predictors of complete negativity across all sequences, even after adjusting for age, serum PSA, PSA density, cancer status, and the interpreting radiologist. These robust associations suggest that acquiring only a single, negative sequence may obviate the need for the additional MRI sequences in a significant proportion of patients, potentially reducing examination times, scanner use, and patient burden.

A considerable proportion of our cohort could potentially benefit from a shortened MRI protocol. Specifically, at least one-third of patients with suspected prostate cancer and approximately 10% of those with known prostate cancer could have concluded their examinations after a single negative sequence. In our dataset, nearly 60% of the patients, regardless of clinical indication, who had a negative T2WI sequence also had negative DWI and DCE sequences. For patients with a negative DWI sequence, this number was even higher, with the T2WI and DCE results also being negative in over 80%.

Both models (T2WI predicting DWI/DCE and DWI predicting T2WI/DCE) performed similarly, as evidenced by their pseudo *R^2^* values. These metrics show that the models explain a substantial portion of the variability in their respective outcomes. However, there are practical considerations for choosing one sequence over the other: T2WI is generally more robust and consistent across institutions, whereas DWI can be more variable and prone to artifacts^(21^,^22)^. Nevertheless, DWI inherently incorporates aspects of T2 information, which may not be fully utilized by a human reader but could be leveraged by an AI model to enhance predictive accuracy^(23^,^24)^. In addition, our results indicate that a negative DWI result is more often associated with negative subsequent sequences than is a negative T2WI result, suggesting that DWI is the more effective predictor.

From a practical standpoint, our findings imply that many examinations could be truncated after acquiring only the T2WI or DWI sequence because the additional sequences would not provide additional diagnostic information. Such a strategy would reduce unnecessary sequences while maintaining diagnostic confidence. At high-volume centers or in resource-limited settings, this could significantly improve scanner availability, reduce patient time in the MRI suite, and enhance overall efficiency.

Implementing a streamlined approach in routine clinical practice poses several challenges. Reliance on real-time radiologist interpretation is neither practical nor efficient, as it could increase workloads and fatigue, potentially compromising diagnostic accuracy^(25)^. Our data also show that depending solely on a single sequence, such as DWI, would lead to incorrect decisions in approximately 10-15% of cases. This level of error is clinically significant, as it risks underdiagnosing or missing clinically important disease^(26)^. It is important to emphasize that this approach would not be appropriate for all patients; a substantial subset will continue to require the full multiparametric MRI protocol to ensure comprehensive assessment. These challenges underscore both the promise and the limitations of abbreviated imaging strategies. Given that this was a hypothesis-generating study, we are encouraged by the results, which suggest that tailoring scan duration to imaging content is feasible. Although this approach is not yet ready for clinical implementation, we believe that further research, particularly research involving AI, will be critical to advancing this strategy. It is possible that AI will play a transformative role in overcoming these limitations. A well-integrated AI model could operate within the imaging workflow to make real-time, data-driven decisions without increasing the cognitive burden of radiologists^(27)^. By identifying subtle or early imaging features that might be imperceptible to the human eye, AI could reduce the error rate associated with early termination of scans, ensuring that patients who need a full examination still receive it. This approach could preserve, or even enhance, diagnostic accuracy while improving efficiency and allowing radiologists to focus on interpretive tasks that are more complex.

It is important to emphasize that a negative MRI result, whether from a comprehensive multiparametric examination or from one truncated after a single sequence, does not guarantee the absence of clinically significant cancer. Patients with negative imaging findings may still harbor disease, including high-grade tumors^(28^,^29)^. Therefore, ongoing surveillance strategies remain essential, including the monitoring of serum PSA levels and other tumor markers, as well as systematic biopsies or follow-up imaging as needed^(30^-^33)^. The intent of our study was not to assess diagnostic accuracy, but rather to demonstrate that, if a single negative sequence can reliably predict a fully negative MRI outcome, then truncating the examination at that point has the potential to streamline the imaging acquisition process.

The retrospective design of our study may have introduced bias, and knowledge of the result of one sequence could have influenced the interpretation of subsequent sequences. Although we attempted to mitigate this by systematically reviewing all sequences categorized as negative, such bias could lead to overestimation of how frequently all sequences are negative.

Looking ahead, the potential impact of this streamlined approach may be particularly pronounced in the context of the rapidly evolving prostate cancer diagnostic pathway. There is growing support for using prostate MRI as a triage tool prior to biopsy in patients with elevated serum PSA^(34^-^36)^, a strategy that could dramatically increase the number of MRI examinations^(37)^. As MRI becomes more widely adopted in this pathway, the ability to shorten scans without compromising diagnostic confidence could be transformative. Such efficiencies would enable centers to accommodate higher volumes of patients, potentially leading to earlier disease detection, improved patient access to care, and reduced overall costs. In this scenario, the approach of limiting the examination to a single predictive sequence when appropriate stands to play a pivotal role in meeting the rising demand for MRI services.

In summary, our study suggests that T2WI or DWI findings can serve as preliminary indicators for the diagnostic yield of subsequent sequences, with DWI appearing to hold a slight advantage. While the accuracy of this approach is not yet sufficient for clinical implementation, these results are promising and support further investigation. Confirmatory studies, particularly involving real-time AI integration, are warranted to enhance prediction performance and establish the practical utility of these abbreviated MRI protocols in clinical practice.
